# The complete mitochondrial genome of the West African honey bee *Apis mellifera adansonii* (Insecta: Hymenoptera: Apidae)

**DOI:** 10.1080/23802359.2019.1693308

**Published:** 2019-12-09

**Authors:** Leigh Boardman, Amin Eimanifar, Rebecca Kimball, Edward Braun, Stefan Fuchs, Bernd Grünewald, James D. Ellis

**Affiliations:** a Honey Bee Research and Extension Laboratory, Entomology and Nematology Department, University of Florida, Gainesville, FL, USA;; b Independent Senior Research Scientist, Industrial District, Easton, MD, USA;; c Department of Biology, University of Florida, Gainesville, FL, USA;; d Institut für Bienenkunde, Polytechnische Gesellschaft, Goethe-Universität Frankfurt am Main, Oberursel, Germany

**Keywords:** Mitogenome, next-generation sequencing, A-lineage honey bee

## Abstract

The complete mitochondrial genome of the West African honey bee *Apis mellifera adansonii* consisted of 13 protein-coding genes, 22 transfer RNA genes, two ribosomal RNA genes, and a control region. It was 16,466 bp and consisted of 84.7% AT nucleotides. This subspecies had a similar mitogenome to those of other southern African honey bees, namely *A. m. scutellata*, *A. m. capensis*, and *A. m. monticola*.

The West African honey bee, *A. m. adansonii* (Latreille, 1804), is a small, yellow honey bee found throughout West Africa, including Nigeria, Burkina Faso, and Congo. Mitochondrial DNA studies on the tRNA-Ile and ND2 gene regions and a microsatellite study both showed that *A. m. adansonii* is closely related to honey bees with which it shares geographic borders: *A. m. scutellata*, *A. m. monticola*, and *A. m. capensis* (Arias and Sheppard 1996; Franck et al. 2001). Using a single gene region, these four subspecies were found to be genetically very similar (Arias and Sheppard 1996). Here, we sequenced the mitochondrial genome of a worker *A. m. adansonii* from the Ruttner Bee Collection at the Bee Research Institute in Oberursel, Germany (Voucher No. 1284, H. Himsel, 1985, Gaya, Niger, 11°52′48N 3°27E). Subspecies identity was confirmed morphometrically and the GenBank accession number is MN585109.

Genomic DNA was extracted, quantified and sequenced (PE-150bp, Illumina Hi-Seq 3000/4000, San Diego, CA) following Eimanifar et al. (2017). Raw sequencing data were quality controlled using FastQC (Andrews 2010). Reads were trimmed with Trimmomatic (Bolger et al. 2014) before mapping was performed in Geneious Prime 2019.0.4 (Biomatters Ltd., Auckland, New Zealand) (Kearse et al. 2012). We followed the stringent mapping practice described in Boardman et al. (2019), using *A. m. capensis* (KX870183) as the reference genome. The assembled mitochondrial genome was annotated using mitos2 (Bernt et al. 2013) and then manually adjusted to *A. m. capensis* in Geneious. Sequences from the 13 protein-coding genes (PCGs) and two ribosomal RNAs (rRNAs) were extracted and manually aligned to other *Apis* sequences in Mesquite version 3.5 (Maddison and Maddison 2018). Phylogenetic analysis was completed using RAxML version 8.2.10 GTRGAMMA model (1000 bootstrap replicates, -f, a option) (Stamatakis 2014) on CIPRES Science Gateway version 3.3 (Miller et al. 2010) and *P*-distances were calculated using PAUP 4.0a (Swofford 2003).

The complete mitogenome of *A. m. adansonii* was 16,466 bp (base composition: 43.2% A, 41.5% T, 5.6% C, and 9.6% G). As expected, the mitogenome has 13 PCGs, 22 transfer RNA (tRNA) genes, two rRNA genes, and one putative control region (CR). The light strand encoded nine PCGs (*nad2*, *co1*, *co2*, *atp8*, *atp6*, *co3*, *nad3*, *nad6*, and *cytb*), with the remaining four (*nad1*, *nad4*, *nad4l*, and *nad5*) on the heavy strand. Nineteen nucleotides were shared between *atp8* and *atp6*. Four start codons were used: ATT (*co2*, *atp8*, *nad5*, *nad4l*, *nad6*, and *nad1*), ATG (*atp6*, *co3*, *nad4*, and *cytb*), ATA (*co1* and *nad3*) and ATC (*nad2*), and all 13 PCGs used a TAA stop codon. The 22 tRNAs varied in length from 62 bp (tRNA-Gln) to 79 bp (tRNA-Thr). The 16S rRNA was 1,327 bp (84.2% AT) and the 12S rRNA was 785 bp (81% AT).

Phylogenetically, the closest subspecies to *A. m. adansonii* were the African honey bees (*A. m. scutellata* hybrid, KJ601784, *P*-distance: 0.00137), *A. m. capensis* (KX870183, *P*-distance: 0.00267), *A. m. scutellata* (KY614238, *P*-distance: 0.00274), and *A. m. monticola* (MF678581, *P*-distance: 0.0032) (Figure 1). Ruttner (1988) suggested that *A. m. adansonii* is similar to *A. m. litorea*, so sequencing this subspecies would further clarify our understanding of sub-Saharan honey bee evolution and diversity.

**Figure 1.**
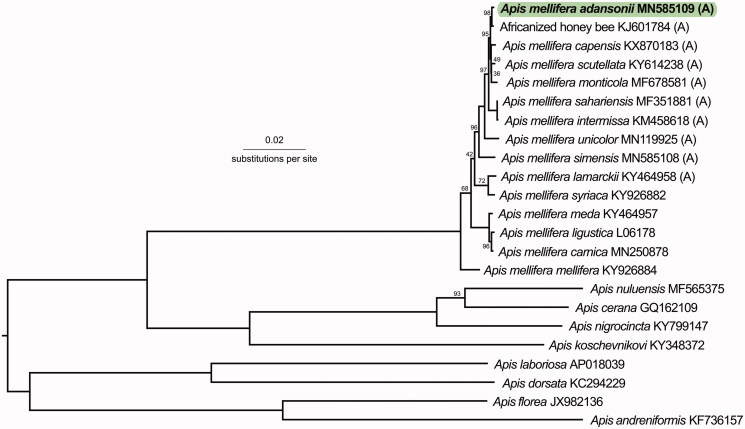
Phylogenetic relationships between *A. m. adansonii* and 22 other *Apis* honey bees (GenBank accession numbers provided). African A-lineage honey bees are shown with (A). The tree is midpoint rooted. Node labels indicate bootstrap values, with unlabeled lineages representing 100%.
